# Petal-like NiS-NiO/G-C3N4 Nanocomposite for High-Performance Symmetric Supercapacitor

**DOI:** 10.3390/mi13122134

**Published:** 2022-12-02

**Authors:** Amira Ben Gouider Trabelsi, Doaa Essam, Fatemah H. Alkallas, Ashour M. Ahmed, Mohamed Rabia

**Affiliations:** 1 Department of Physics, College of Science, Princess Nourah bint Abdulrahman University, P.O. Box 84428, Riyadh 11671, Saudi Arabia; 2 Nanophotonics and Applications Lab, Physics Department, Faculty of Science, Beni-Suef University, Beni-Suef 62514, Egypt; 3 Nanomaterials Science Research Laboratory, Chemistry Department, Faculty of Science, Beni-Suef University, Beni-Suef 62514, Egypt; 4 Physics Department, College of Science, Imam Mohammad Ibn Saud Islamic University (IMSIU), Riyadh 11623, Saudi Arabia

**Keywords:** NiO, NiS, G-C3N4, porous materials, specific capacitance, electrolytes

## Abstract

Graphitic carbon nitride (G-C3N4) and NiS-NiO/G-C3N4 nanocomposite have been synthesized via combustion and hydrothermal techniques, respectively. The chemical and morphological properties of these materials were confirmed using different analytical methods. SEM confirms the formation of G-C3N4 sheets containing additional petal-like shapes of NiS-NiO nanoparticles. The electrochemical testing of NiS-NiO/G-C3N4 symmetric supercapacitors is carried out from 0.6 M HCl electrolyte. Such testing includes charge/discharge, cyclic voltammetry, impedance, and supercapacitor stability. The charge/discharge time reaches 790 s at 0.3 A/g, while the cyclic voltammetry curve forms under a high surface area. The produced specific capacitance (C_S_) and energy density values are 766 F/g and 23.55 W.h.kg^−1^, correspondingly.

## 1. Introduction

Supercapacitors progress last decay particularly between energy storage devices rises owing to their high ability to provide sustainable energy [1,2,3]. Supercapacitors become an equivalent source to the batteries owing to their easy and fast charging time, which contrasts with their high discharging time. Such a device could be classified into three categories: an electric double-layer capacitor, hybrid capacitor, and pseudocapcitance capacitor [4,5]. The carbon supercapacitor is called an electric double-layer capacitor, in which the charges are stored as a double layer between the carbon and electrolyte. A pseudocapcitoance supercapacitor, however, is based on a material, i.e., oxides, sulfides, or nitrides, owing to a high ability to redox reactions inside the capacitor. However, the different existing supercapacitor types are limited by their small energy power [6]. Thus, the hybrid supercapacitor is the most promising type that brings together the entire advantages of the previous types. Indeed, this is assigned to its particular construction grouping of both carbon and normal materials that possess the excellent capability of redox and double-layer storage behavior at the same time [7]. 

In this regard, the choice of a base material that has a great storage ability plays a major role in enhancing a supercapacitor. Thus, utilizing a composite material will provide additional advantages emerging from the combination of the electric conductivity properties of various materials in the compound. Moreover, oxide and sulfide materials with a great surface area also provide an extra active site for charge storage [6]. In addition, MXene shows high storage ability for supercapacitor and battery applications [8,9,10,11]. 

Graphitic carbon nitride (G-C3N4) is a promising material for supercapacitors due to its magnificent properties of charge storage represented in the high surface area and charge-chemical durability. This material could be prepared with a high mass amount under the combustion of some organic materials rich in nitrogen, such as urea and thiourea. This explains its chemical structure of tris-triazine (C6N7) connected through a ternary amino group [12].

Earlier studies were carried out on G-C3N4 for supercapacitor applications [9,10]. Santos et al. studied the synthesis of CoO and CuO on G-C3N4, where a specific capacitance (CS) was found equal to 84.28 F/g at 0.5 A g [13]. Kuila et al. considered Gd decorated on G-C3N4 for supercapacitor applications. Herein, the produced capacitance value was 2.59 mF cm^−2^ at 10 mV s^–1^ [14]. Rani et al. examined similarly CoFe2O4/G-C3N4 for supercapacitor application, and a CS = 524 F/g at 2 mV/s was found [15]. Furthermore, G-C3N4/graphene was prepared as an electrode for the supercapacitor, in which the Cs value was 265 F/g [16]. On the other hand, a G-C3N4/carbon nanotube was applied as a supercapacitor from polyvinyl alcohol/H2SO4 electrolyte; the produced CS value was about 148 F/g [17]. Also, G-C3N4/activated carbon from 1 M Na2SO4 was inspected for supercapacitor, and C_S_ = 266 F/g was obtained [18]. Additionally, Ni(OH)_2_/G-C3N4 was prepared using the hydrothermal method, for which the produced C_S_ value was 555 F/g [19]. 

In this work, G-C3N4 is prepared via urea combustion in ambient air, then a NiS-NiO/G-C3N4 petal-like nanocomposite was synthesised using a hydrothermal method. All the chemical and morphological analyses confirmed the composite properties. Such a composite was used as an electrode for the symmetric supercapacitor in energy storage. The electrochemical testing is carried out from a 0.6 M HCl electrolyte: charge/discharge, cyclic voltammetry, impedance, and supercapacitor stability. The specific capacitance, energy density, and lifetime is calculated for this promising supercapacitor. The prepared supercapacitor is a promising result, represented in the produced CS and E values. Soon, our team will work on the design of a prototype of this capacitor for commercial applications.

## 2. Experimental Section

### 2.1. Materials

HCl (36%), urea (99.99%), and thiourea (99.9%,) were obtained from Piochem co., Cairo, Egypt. Nickel sulfate (NiSO_4_), ammonium persulfate ((NH_4_)_2_S_2_O_8_), graphite powder, and ammonia (NH_4_OH) were obtained from El Naser Chemical Co., Cairo, Egypt. Sulfonated tetrafluoroethylene dissolved in methanol (5 wt.%) was obtained from Sigma-Aldrich, St. Louis, MO, USA.

### 2.2. Preparation of NiS-NiO/G-C3N4 Nanocomposite

The preparation of G-C3N4 was carried out through the combustion of material rich with nitrogen (urea). A 10 g sample of urea was combusted well at 550 °C for 2 h in ambient air. This high temperature decomposes the urea to form G-C3N4.

**Figure 1:**
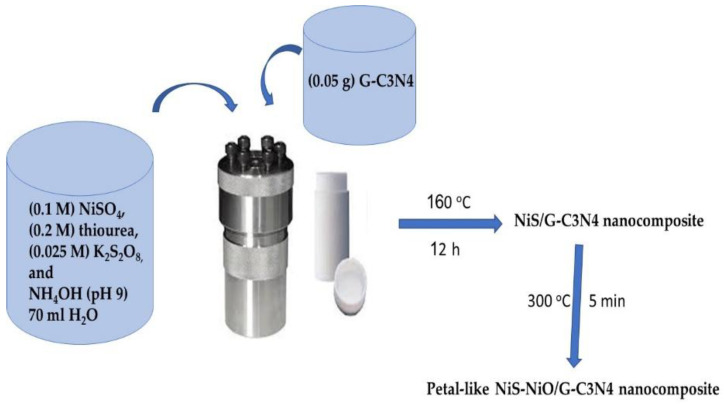
The schematic preparation for the Petal-like NiS-NiO/G-C3N4 nanocomposite.

NiS-NiO/G-C3N4 nanocomposite synthesis was obtained through dissolving (0.1 M) NiSO_4_, (0.2 M) thiourea, and (0.025 M) K_2_S_2_O_8_ in 70 mL H_2_O by using an ultrasonic device. NH_4_OH is used as a basic medium for clear-dissolving NiSO_4_, in which the pH solution becomes 9. Then, 0.05 g C3N4 is suspended in H_2_O using the ultrasonic device. Thenceforth, G-C3N4 is poured over the solution in an autoclave, and the hydrothermal reaction is carried out at 160 °C for 12 h. The produced powder was cleaned and dried at 60 °C for 6 h. Herein, the combustion process is carried out at 300 °C for 5 min, in which the nanocomposite NiS-NiO/G-C3N4 is formed. The schematic preparation for the Petal-like NiS-NiO/G-C3N4 nanocomposite is mentioned in Figure 1.

### 2.3. Synthesis and Testing of the Prepared Symmetric Supercapacitor

The NiS-NiO/G-C3N4 composite was synthesized using (0.045 g) and (0.005 g) of graphite mixed with sulfonated tetrafluoroethylene and 0.75 mL of C_2_H_5_OH. For the preparation of two symmetric electrodes, 0.003 g paste is loaded on an Au plate (1 cm^2^). These two electrodes are combined through a separator filter paper wetted with 0.5 M HCl, then tested using an electrochemical workstation (CHI660E), in which the charge/discharge, stability, and impedance are measured. The specific capacitance (C_S_) and power energy (E) of the supercapacitor are also calculated. 

### 2.4. Characterization

The different structures and morphological properties of the prepared materials were inspected using several characterization techniques. X-ray diffraction (X’Pert Pro, Almelo, The Netherlands) and X-ray photoelectron spectroscopy are used to confirm the chemical structure of the prepared materials. The morphologies were determined via a scanning electron microscope, SEM, (ZEISS SUPRA 55 VP, Column, Oberkochen, Germany), and transmitted electron microscope (TEM), (JEOL JEM-2100, Oberkochen, Germany).

## 3. Results and Discussion

### 3.1. Analyses

**Figure 2:**
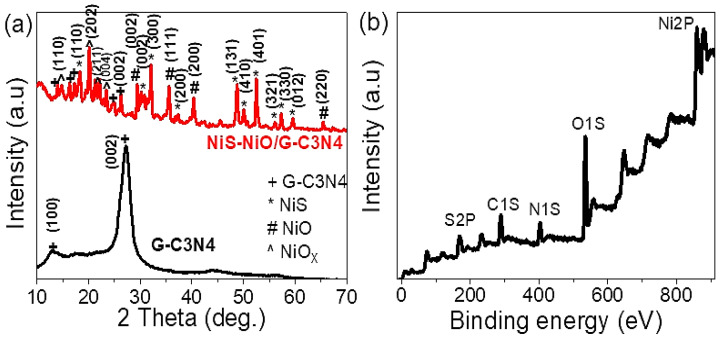
(**a**) XRD and (**b**) XPS for NiS-NiO/G-C3N4 composite.

The XRD pattern of the obtained graphitic-like layer structures of G-C3N4 and NiS-NiO/ G-C3N4 has been investigated (see Figure 2a). For G-C3N4, two distinct diffraction peaks have been located: a small diffraction peak assigned to (100) at 13.03° is attributed to the in-planar structural packing motif with a separation of 0.679 nm, while a strong other peak located at 27.20° corresponds to the (002) peak of the interlayer d-spacing of 0.327 nm [20,21]. The two peaks at 13.03° and 27.20° are matched with the previous studies (JCPDS 87–1526 card) [20] and (JCPDS) 87–1526 for g-C3N4 [21]. The composite sample exhibits diffraction peaks corresponding to both G-C3N4, NiS, NiO, and NiO_X_.

The G-C3N4 material has a small shift in peak at 27.20°, moreover, there is the appearance of additional peaks in the composite, confirming the effect of inorganic materials during the composite formation. NiS nanomaterials has ten peaks located at 18.18, 30.30, 32.10, 37.30, 48.87, 50.30, 52.57, 56.19, 57.60, 59.74° for the growth directions (110), (002), (300), (200), (131), (410), (401), (321), (330), and (012), respectively. Most of these peaks matched with previous studies (JCPDS 12-0041) [22]. However, NiO displayed four characteristic peaks positioned at 29.35, 35.88, 40.36, and 65.55° for the growth direction (002), (111), (200), and (220), which agree with previous studies (JCPDS, No. 04-0835) [23]. Moreover, additional peaks have been observed, confirming the formation of additional oxides for Ni; these peaks are positioned at 14.87, 20.23, 21.80, and 23.52° [24]. 

Figure 2b illustrates the X-ray photoelectron spectroscopy (XPS) of the prepared composite. This will confirm its chemical composition and structure. Indeed, several elements have been distinguished in the sample, such as O, Ni, N, C, and S (see Table 1). Indeed, O1s spectra are located at 534 eV, the element source of the NiO and NiO_X_ materials. The C 1s XPS spectra originated from the utilized pure G-C3N4. Several bonds, such as C-C, C-NH_2_, and N-C=N bonding at about 284, 286, and 288 eV, respectively, have been also identified. The C-C XPS peak slightly shifts to higher binding energy as the signature of the chemical bonding between G-C3N4 and NiS-NiO. The N1s strong peak centering at 402.66 eV is owing to the G-C3N4 material after forming a composite with the additional Ni materials. Furthermore, the Ni2p peak at 858.91 eV is assigned Ni^2+^ in Ni^X+^ species due to the easy NiO oxidation in the air. Also, an S2p peak located at 167 eV could be assigned to sulfur in the NiS bonding, revealing the high binding between NiS-NiO and G-C3N4 during the composite formation.

**Figure 3:**
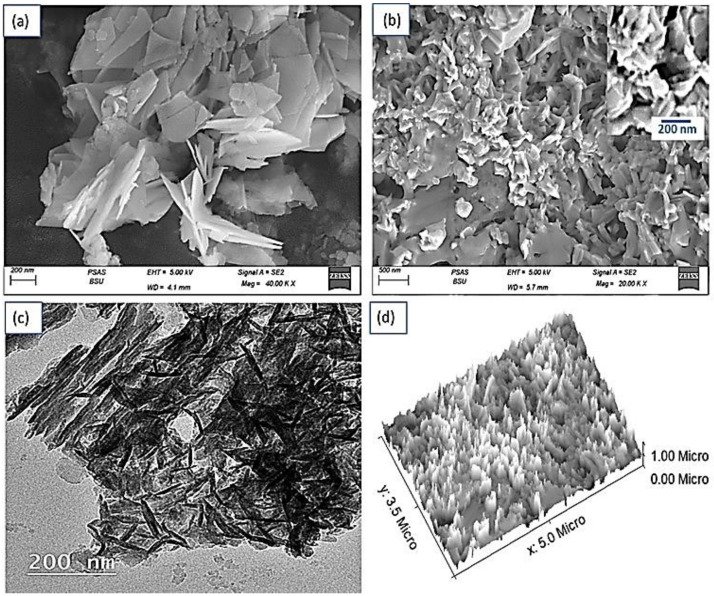
SEM of (**a**) G-C3N4 and (**b**) NiS-NiO-G C3N4 composite. (**c**) TEM and (**d**) theoretical image for cross-section and roughness of NiS-NiO/G-C3N4 composite.

The morphological and structural analysis of the samples was based on the correlation between SEM and TEM measurements. Figure 3 represents SEM images of (a) G-C3N4 and (b) NiS-NiO-G C3N4 composite. It is seen that both images reveal a thin sheet structure. These nanosheets have an average width and length of 80 nm and 170 nm. After the composite formation, the morphology is enhanced clearly, which appears clearly in the nanopetal-like shapes with an average particle size of 150 nm. The high porosity motivates the application of these nanomaterials as a great electrical device for energy storage.

Indeed, this was clearly distinguished through the TEM image of the G-C3N4 illustrating flat irregular shape sheets with wrinkles (see Figure 3c). The dark area for the G-C3N4 sheets confirms the NiS-NiO loaded on the G-C3N4. These analyses confirm the nanomaterials of the composite with a great homogeneity around the G-C3N4 sheets. Figure 3d represents the cross-section and roughness of the composite using the Gwydion program. The obtained theoretical image confirms the unique behavior of the porous structure of the composite. 

### 3.2. The Electrochemical Testing for the Prepared Supercapacitor

**Figure 4:**
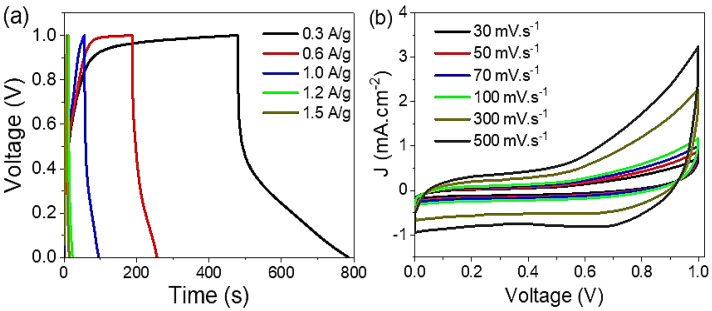
(**a**) Charge/discharge and (**b**) Cyclic voltammetry for symmetric supercapacitor based on NiS-NiO/G-C3N4 nanocomposite under HCl electrolyte.

The symmetric supercapacitor based on the two electrodes NiS-NiO/G-C3N4 nanocomposite is measured using the electrochemical workstation (CHI660E) in a potential window of 0.0 to 1.0 V at 25 °C (see Figure 4a). The measurements are carried out between the two plates, in which the paste (0.003 g) is loaded on Au metals, and filter paper wetted with 0.5 M HCl is used as a separator. The charge/discharge measurements are carried out in a current density from 0.3 to 1.5 A/g, in which, with increasing the current density, the discharge time decreases. This confirms the effect of current density values on the capacitance of the prepared supercapacitor associated with raising the input current. The supercapacitor does not have the efficiency time to carry charges on its plates [25,26]. The values of discharge time at 0.3 and 0.6 A/g are 790 and 260 s, respectively. Using HCl as an electrolyte is related to the high amount of H^+^ ions inside the acid with its great mobility; this works as a perfect electrolyte for charge storage and the generation of pseudocapacitance properties [27,28] inside the NiS-NiO/G-C3N4 nanocomposite.

On the other hand, Figure 4b shows the cyclic voltammetry curves for supercapacitors under various scan rates of 30 to 500 mV.s^−1^. The current density (J) values increase from 0.7 to 3.3 mA.cm^−2^ with increasing the scan rate, respectively, at 1.0 V. The rise of the J value is related to the diffusion of the H^+^ ions inside the paste with a scan rate that causes an increase in the oxidation and reduction current and then the area under the curves [29,30]. 

**Figure 5:**
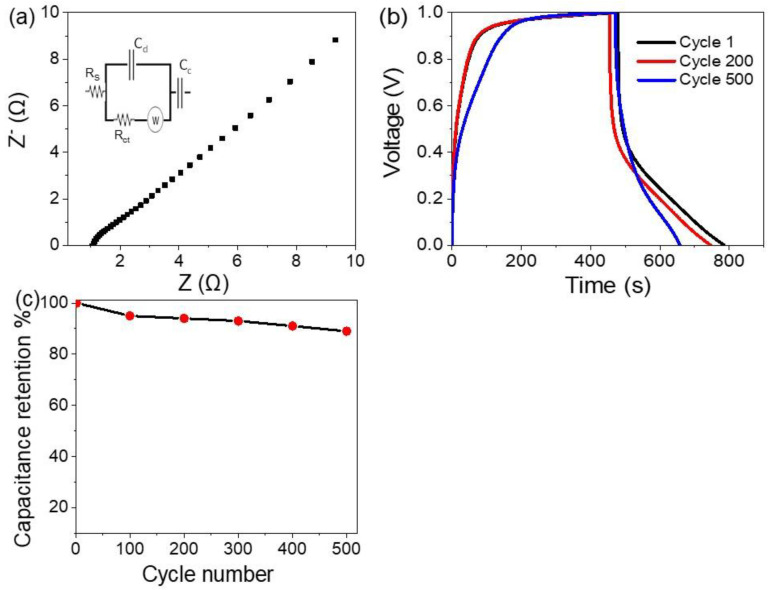
(**a**) The impedance (**b**) stability, (**c**) capacitance retention of the prepared supercapacitor.

The Nyquist plot for the symmetric NiS-NiO/G-C3N4 nanocomposite supercapacitors is represented through the real (Z) and imaginary (Z^−^) relation, as shown in Figure 5a. The plot represents the facilitates of charge transfer between the electrode and electrolyte (0.5 M HCl). The relational small-diameter semicircle images the easy motion of charges through the supercapacitor electrodes [31]. Rundle’s cell is inserted in Figure 5a, in which Rs, Rct, Cd, and W are assigned to the resistances of the solution, charge transfer, capacitor, and Warburg impedance [32,33], respectively. The diameter of the semicircle is equal to 0.78 Ω, which is very small and represents the charge transfer between the electrode and electrolyte interface. This demonstrates the ability of the electrodes in transferring charge, which consequently establishes the great capacitance of the supercapacitor.

The supercapacitor stability and reproducibility are represented in Figure 5b, in which the charge/discharge behavior is repeated for 500 cycles. The stability is measured in 0.5 M HCl with a current density of 0.3 A/g. The capacitance retention for the supercapacitor is mentioned in Figure 5c, in which the supercapacitor has a capacitance retention of 89% till 500 cycles. 

The performance of the supercapacitor is determined through the specific capacitance (*C_S_*) and the Energy density (*E*). The *C_S_* parameter is calculated from Equation (1) [34,35]. The *C_S_* parameter depends on the current *I*, Δt, ΔV, and m, which represent the current, discharge time, potential window, and loaded mass, respectively. From this figure, the value of *C_S_* is 766 F/g. Furthermore, the E parameter depends on *C_S_* and square values of the potential window (Equation (2), while the E value is 23.55 W.h.kg^−1^. Moreover, the power density (*P*) is calculated through Equation (3), in which its value is 173.77 W.kg^−1^.

(1) $$C_{s}=4I.\Delta t/\;\Delta V.m$$

(2) $$E=0.5C_{s}.\;\left(V_{max\;}^{2}-V_{min\;\;}^{2}\right)/3.6$$

(3)                  P=3600E/Δt

**Figure 6:**
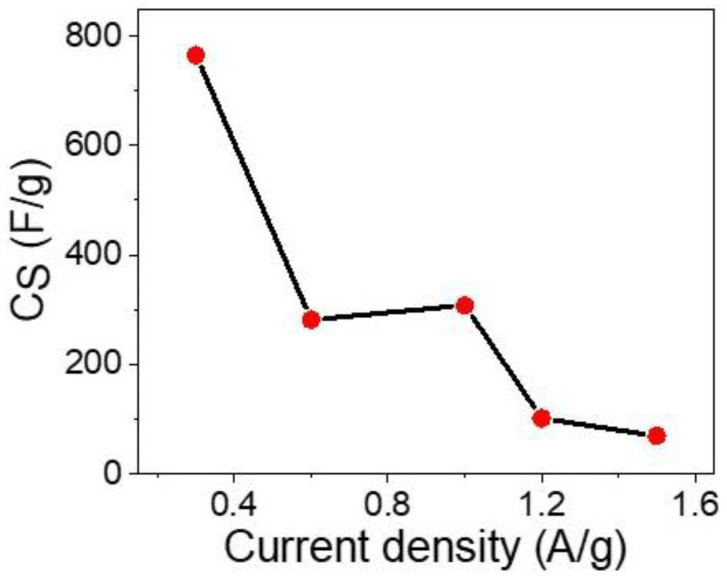
Rate capability of the prepared supercapacitor.

The rate capability (specific capacitance vs. current density) is mentioned in Figure 6. This figure gives an indication of the enhancement of the C_S_ values of the supercapacitor under lower current density values, in which the C_S_ values increase from 71 to 766 F/g, with a decreasing of the current density values from 1.5 to 0.3 A/g, respectively. 

## 4. Conclusions

NiS-NiO/G-C3N4 nanocomposite has been prepared using the hydrothermal method, in which the composite is based on G-C3N4 prepared using the combustion process in ambient air. From the morphological analyses, G-C3N4 has a nanosheet structure. After composite formation, petal-like nanomaterials are formed. The XRD and XPS analyses confirmed the presence of NiS, NiO, NiO_X_, and G-C3N4 materials. The prepared composite, NiS-NiO/G-C3N4, was applied as a paste for the symmetric supercapacitor. The electrochemical measurements (charge/discharge, cyclic voltammetry, impedance, and stability) were carried out from 0.6 M HCl electrolyte. The discharge time reaches 790 s at 0.3 A/g. The C_S_ and energy density values are 766 F/g and 23.55 W.h.kg^−1^, respectively. These properties qualify the prepared supercapacitor for commercial application, soon.

## Figures and Tables

**Table 1:** Peak location and atomic percent for each element in the composite NiS-NiO/G-C3N4 calculated from XPS analysis.

Element	Peak Location (eV)	Atomic %
O1s	534.6	37.67
Ni2p	858.9	6.42
N1s	402.6	13.29
C1s	288.6	32.14
S2p	167.2	10.48

## Data Availability

The data that support the findings of this study are available from the corresponding author upon reasonable request.

## References

[1] Abdelazeez A.A.A., Trabelsi A.B.G., Alkallas F.H., Rabia M. (2022). Successful 2D MoS2 Nanosheets Synthesis with SnSe Grid-like Nanoparticles: Photoelectrochemical Hydrogen Generation and Solar Cell Applications. Solar Energy.

[2] Shaban M., Ali S., Rabia M. (2019). Design and application of nanoporous graphene oxide film for CO_2_, H_2_, and C_2_H_2_ gases sensing. J. Mater. Res. Technol..

[3] Elsayed A.M., Rabia M., Shaban M., Aly A.H., Ahmed A.M. (2021). Preparation of hexagonal nanoporous Al_2_O_3_/TiO_2_/TiN as a novel photodetector with high efficiency. Sci. Rep..

[4] Mai L.Q., Minhas-Khan A., Tian X., Hercule K.M., Zhao Y.L., Lin X., Xu X. (2013). Synergistic interaction between redox-active electrolyte and binder-free functionalized carbon for ultrahigh supercapacitor performance. Nat. Commun..

[5] Krishnamoorthy K., Pazhamalai P., Mariappan V.K., Nardekar S.S., Sahoo S., Kim S.J. (2020). Probing the energy conversion process in piezoelectric-driven electrochemical self-charging supercapacitor power cell using piezoelectrochemical spectroscopy. Nat. Commun..

[6] Mai L.Q., Yang F., Zhao Y.L., Xu X., Xu L., Luo Y.Z. (2011). Hierarchical MnMoO4/CoMoO4 heterostructured nanowires with enhanced supercapacitor performance. Nat. Commun..

[7] Chahal P., Madaswamy S.L., Lee S.C., Wabaidur S.M., Dhayalan V., Ponnusamy V.K., Dhanusuraman R. (2022). Novel manganese oxide decorated polyaniline/graphitic carbon nitride nanohybrid material for efficient supercapacitor application. Fuel.

[8] Javed M.S., Mateen A., Ali S., Zhang X., Hussain I., Imran M., Shah S.S.A., Han W. (2022). The Emergence of 2D MXenes Based Zn-Ion Batteries: Recent Development and Prospects. Small.

[9] Javed M.S., Shah S.S.A., Najam T., Siyal S.H., Hussain S., Saleem M., Zhao Z., Mai W. (2020). Achieving high-energy density and superior cyclic stability in flexible and lightweight pseudocapacitor through synergic effects of binder-free CoGa_2_O_4_ 2D-hexagonal nanoplates. Nano Energy.

[10] Javed M.S., Zhang X., Ali S., Mateen A., Idrees M., Sajjad M., Batool S., Ahmad A., Imran M., Najam T. (2022). Heterostructured bimetallic–sulfide@layered Ti3C2Tx–MXene as a synergistic electrode to realize high-energy-density aqueous hybrid-supercapacitor. Nano Energy.

[11] Javed M.S., Shaheen N., Hussain S., Li J., Shah S.S.A., Abbas Y., Ahmad M.A., Raza R., Mai W. (2019). An ultra-high energy density flexible asymmetric supercapacitor based on hierarchical fabric decorated with 2D bimetallic oxide nanosheets and MOF-derived porous carbon polyhedra. J. Mater. Chem. A.

[12] Zheng Z.X., Wang M., Shi X.Z., Wang C.M. (2019). Palladium Nanoparticles/Graphitic Carbon Nitride Nanosheets-Carbon Nanotubes as a Catalytic Amplification Platform for the Selective Determination of 17α-ethinylestradiol in Feedstuffs. Sci. Rep..

[13] Santos R.S., Suresh Babu R., Devendiran M., Haddad D.B., de Barros A.L.F. (2022). Facile synthesis of transition metal (M = Cu, Co) oxide grafted graphitic carbon nitride nanosheets for high performance asymmetric supercapacitors. Mater. Lett..

[14] Kumar Kuila S., Ghorai A., Midya A., Sekhar Tiwary C., Kumar Kundu T. (2022). Chemisorption of gadolinium ions on 2D-graphitic carbon nitride nanosheet for enhanced solid-state supercapacitor performance. Chem. Phys. Lett..

[15] Rani B., Nayak A.K., Sahu N.K. (2021). Electrochemical supercapacitor application of CoFe_2_O_4_ nanoparticles decorated over graphitic carbon nitride. Diam. Relat. Mater..

[16] Lin R., Li Z., Abou El Amaiem D.I., Zhang B., Brett D.J.L., He G., Parkin I.P. (2017). A general method for boosting the supercapacitor performance of graphitic carbon nitride/graphene hybrids. J. Mater. Chem. A.

[17] Lu C., Chen X. (2020). Carbon nanotubes/graphitic carbon nitride nanocomposites for all-solid-state supercapacitors. Sci. China Technol. Sci..

[18] Pilathottathil S., Kavil J., Shahin Thayyil M. (2022). Boosting ion dynamics by developing graphitic carbon Nitride/Carbon hybrid electrode materials for ionogel supercapacitor. Mater. Sci. Eng. B.

[19] Shi L., Zhang J., Liu H., Que M., Cai X., Tan S., Huang L. (2015). Flower-like Ni(OH)_2_ hybridized g-C_3_N_4_ for high-performance supercapacitor electrode material. Mater. Lett..

[20] Azizi-Toupkanloo H., Karimi-Nazarabad M., Shakeri M., Eftekhari M. (2019). Photocatalytic mineralization of hard-degradable morphine by visible light-driven Ag@g-C3N4 nanostructures. Environ. Sci. Pollut. Res..

[21] Kumar A., Kumar P., Joshi C., Manchanda M., Boukherroub R., Jain S.L. (2016). Nickel Decorated on Phosphorous-Doped Carbon Nitride as an Efficient Photocatalyst for Reduction of Nitrobenzenes. Nanomaterials.

[22] Ma Z., Yuan X., Zhang Z., Mei D., Li L., Ma Z.F., Zhang L., Yang J., Zhang J. (2015). Novel Flower-like Nickel Sulfide as an Efficient Electrocatalyst for Non-aqueous Lithium-Air Batteries. Sci. Rep..

[23] Wei Z., Qiao H., Yang H., Zhang C., Yan X. (2009). Characterization of NiO nanoparticles by anodic arc plasma method. J. Alloys Compd..

[24] Rakshit S., Ghosh S., Chall S., Mati S.S., Moulik S.P., Bhattacharya S.C. (2013). Controlled synthesis of spin glass nickel oxide nanoparticles and evaluation of their potential antimicrobial activity: A cost effective and eco friendly approach. RSC Adv..

[25] Atta A., Abdelhamied M.M., Essam D., Shaban M., Alshammari A.H., Rabia M. (2021). Structural and physical properties of polyaniline/silver oxide/silver nanocomposite electrode for supercapacitor applications. Int. J. Energy Res..

[26] Gamal A., Shaban M., BinSabt M., Moussa M., Ahmed A.M., Rabia M., Hamdy H. (2022). Facile Fabrication of Polyaniline/Pbs Nanocomposite for High-Performance Supercapacitor Application. Nanomaterials.

[27] Hao J., Huang Y., He C., Xu W., Yuan L., Shu D., Song X., Meng T. (2018). Bio-templated fabrication of three-dimensional network activated carbons derived from mycelium pellets for supercapacitor applications. Sci. Rep..

[28] Ramadan M., Abdellah A.M., Mohamed S.G., Allam N.K. (2018). 3D Interconnected Binder-Free Electrospun MnO@C Nanofibers for Supercapacitor Devices. Sci. Rep..

[29] Sayyah E.S.M., Shaban M., Rabia M. (2018). A sensor of m-cresol nanopolymer/Pt-electrode film for detection of lead ions by potentiometric methods. Adv. Polym. Technol..

[30] Sayyah S.M., Shaban M., Rabia M. (2018). Electropolymerization of m-Toluidin on Platinum Electrode from Aqueous Acidic Solution and Character of the Obtained Polymer. Adv. Polym. Technol..

[31] Mohamed F., Rabia M., Shaban M. (2020). Synthesis and characterization of biogenic iron oxides of different nanomorphologies from pomegranate peels for efficient solar hydrogen production. J. Mater. Res. Technol..

[32] Jones P.K., Stimming U., Lee A.A. (2022). Impedance-based forecasting of lithium-ion battery performance amid uneven usage. Nat. Commun..

[33] Qu D., Ji W., Qu H. (2022). Probing process kinetics in batteries with electrochemical impedance spectroscopy. Commun. Mater..

[34] Yu J., Fu N., Zhao J., Liu R., Li F., Du Y., Yang Z. (2019). High Specific Capacitance Electrode Material for Supercapacitors Based on Resin-Derived Nitrogen-Doped Porous Carbons. ACS Omega.

[35] Naveed ur Rehman M., Munawar T., Nadeem M.S., Mukhtar F., Maqbool A., Riaz M., Manzoor S., Ashiq M.N., Iqbal F. (2021). Facile synthesis and characterization of conducting polymer-metal oxide based core-shell PANI-Pr2O–NiO–Co3O4 nanocomposite: As electrode material for supercapacitor. Ceram. Int..

